# Prophylactic McCall Culdoplasty by a Vaginal Approach during Mini-Laparoscopic Hysterectomy

**DOI:** 10.1155/2019/8047924

**Published:** 2019-05-19

**Authors:** Servet Gencdal, Emine Demirel, Zeynep Soyman, Sefa Kelekci

**Affiliations:** ^1^Izmir Katip Celebi University, Atatürk Education and Research Hospital, Department of Obstetrics and Gynecology, Izmir, Turkey; ^2^Istanbul Education and Research Hospital, Department of Obstetrics and Gynecology, Istanbul, Turkey

## Abstract

**Background:**

In gynecological surgery, one particular area of concern after hysterectomy is the risk of developing an enterocele or vaginal apical prolapse. The aims of this study were to evaluate the safety and efficacy of prophylactic McCall culdoplasty (MC) performed during mini-total laparoscopic hysterectomy (mini-TLH), as well as to compare the differences in apical support, total vaginal length (TVL), and sexual function at one and two years postoperatively.

**Methods:**

Data were retrospectively reviewed for all women who underwent mini-TLH and mini-TLH + MC at a tertiary care center between August 2012 and January 2016 were from the hospital database. There were 18 women who underwent mini-TLH + MC and were considered as the study group, while 20 women who were treated with mini-TLH were considered as the control group. The primary outcome measures were the differences in apical support and TVL and impact on sexual function.

**Results:**

After mini-TLH + MC, the apical vaginal support difference was improved by 2.2 cm. The mean difference in C point was 1.03 cm in the mini-TLH group, which was not significant at two years after the operation. The vaginal vault descent at two years after operation was more prominent in the mini-TLH group than the mini-TLH + MC groups. Apical support changes at two years after the operation were more prominent in the mini-TLH group (0.5 ± 0.6 cm) than the mini-TLH + MC group (1.9 ± 1.2 cm).

**Conclusion:**

Prophylactic MC by a vaginal approach during mini-TLH is safe, satisfactory, and efficient for apical support without severe morbidity.

## 1. Introduction

In gynecological surgery, one particular area of concern after hysterectomy is the risk of developing an enterocele or vaginal apical prolapse. Suspension of the vaginal apex to the uterosacral ligaments or to the sacrospinous ligaments at the time of vaginal hysterectomy is the mainstay for the prevention of posthysterectomy vaginal vault prolapse [1]. An increased likelihood of performing an apical support procedure has been shown to be associated with increased age, hospital type (urban vs. teaching), hospital bed size (large and medium), and hysterectomy type (vaginal and laparoscopically assisted vaginal) in hysterectomies performed without diagnosis of prolapse [2].

Among all hysterectomy methods, the frequency of performing total laparoscopic hysterectomy (TLH) has recently increased worldwide. However, concomitant preventive measures for vaginal vault prolapse have not increased at the same rate [3]. Laparoscopic hysterectomy is firmly established in current gynecological practice, but ongoing efforts are now focused on developing strategies to improve the support of the vaginal vault.

The modified McCall culdoplasty (MC) is a relatively simple procedure that is performed after the removal of the uterus and cervix from the apex of the vagina, where the angles of the vagina are attached to their respective uterosacral ligaments, and the cul-de-sac is surgically obliterated for support postoperatively [4, 5]. Although commonly performed, there are limited or no data describing the outcomes for MC [6] after mini-total laparoscopic hysterectomy (mini-TLH). The aims of the present study are to evaluate the safety and efficacy of performing prophylactic MC vaginally before closing the vaginal cuff during mini-TLH, as well as to compare the differences in apical support, total vaginal length (TVL), and sexual function at one and two years postoperatively in mini-TLH with and without MC.

## 2. Materials and Methods

Data regarding all women who underwent mini-TLH and mini-TLH + MC at a tertiary care center between August 2012 and January 2016 were retrospectively reviewed from the hospital database. Institutional review board approval was obtained for this study (2016/ TP18). the inclusion criteria were as follows: patients who had complete medical records, underwent mini-TLH + MC or mini-TLH for benign diseases, were operated on by the senior surgeon, had at least two years of follow-up, and had no symptomatic or asymptomatic pelvic organ prolapse beyond stage 1 (pelvic organ prolapse-quantitative (POP-Q) classification) before mini-TLH. We excluded all patients with an obliterated cul-de-sac, postmenopause, obesity, polycystic ovarian disease, connective tissue disorders, and previous surgery for pelvic organ prolapse. The identified women were divided into two groups according to whether they underwent mini-TLH or mini-TLH + MC. All patients were informed of these two procedures' differences and probable advantages preoperatively. Surgeries were executed in accordance with patient preference, and their informed consent was obtained.

Operative laparoscopy was performed under general anesthesia in all women. A pneumoperitoneum was created using a Veress needle, and then a 0-degree 5-mm laparoscope (Karl Storz, Tuttlingen, Germany) was introduced through the umbilicus. Two or three 3-mm ancillary trocars were inserted under direct visualization in the lower abdomen. One 3-mm trocar was always inserted in the midline approximately 3 cm above the symphysis. The other trocars were inserted under laparoscopic visualization laterally to the medial umbilical ligament. The mini-TLH was performed in the manner described previously [7, 8].

**Figure 1:**
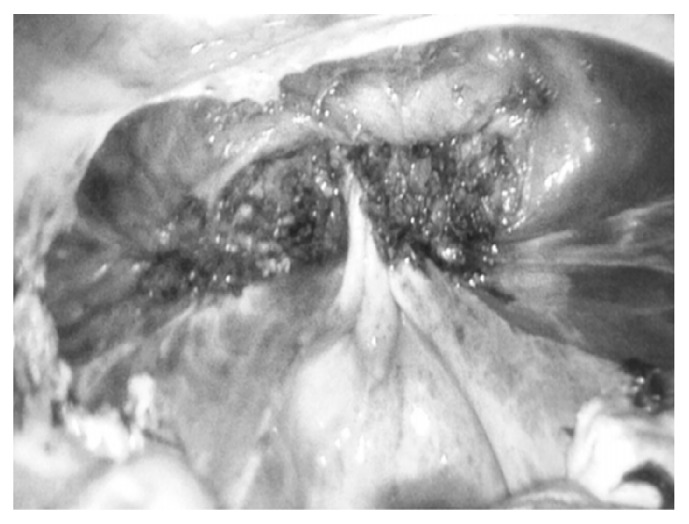
Endoscopic view of the vaginal apex after tying of the modified McCall culdoplasty Suture.

In addition to mini-TLH, bilateral salpingectomy was performed. In general, the procedure was performed most commonly using bipolar coagulation and scissors. After removal of the uterus, adnexa, or both through the vagina, the vaginal cuff was closed laparoscopically with a running delayed absorbable suture (No.1 Vicryl; Ethicon, Livingston, UK) in only the mini-TLH group. In the mini-TLH + MC group, vaginal cuff closure and MC were performed with No.1 Vicryl by a vaginal approach after removal of the uterus (Figure 1) [9].

The demographic parameters (age, gravidity, parity, and body mass index), all values of total vaginal length (TVL) and apical support, satisfaction rate, blood loss during the operation, duration of the operation, and complications were extracted from medical charts. The primary outcome measures were the differences in apical support and TVL, as well as the impact on sexual function. The apical support was evaluated via measurement of the C point at one and two years after surgery. The TVL was examined according to the POP-Q classification preoperatively at one and two years postoperatively. The differences were compared within and between groups. The evaluation of apical support via measurement of the C point and TVL were assessed by the same author according to the POP-Q classification who was blinded to the groups.

In both groups, the patients were evaluated with the Female Sexual Function Index (FSFI) questionnaire at one and two years after surgery. The FSFI questionnaire is a self-reporting measure of sexual function with 19 questions dealing with 6 components of female sexual function: desire, arousal, lubrication, orgasm, satisfaction, and pain [10, 11]. The index was adapted to Turkish population by Aygin et al. [12]. The satisfaction rate was defined as the percentage of women without sexual dysfunction according to the FSFI questionnaire. The patients were not evaluated with the FSFI questionnaire before surgery. The secondary outcome measures included blood loss during the operation, duration of the operation, and complications.

Data were analyzed with SPSS software (SPSS version 15.0 for Windows, IBM). The mean, median, standard deviation, lowest and highest frequency, and ratio values were used as complimentary statistical data. Parametric tests were applied to data with a normal distribution, and nonparametric tests were applied to data with a nonnormal distribution. Frequencies with percentages and medians with ranges were used to describe categorical variables, whereas chi-squared tests compared the counts of categorical data. All differences associated with a chance probability of ≤ 0.05 were considered statistically significant.

## 3. Results

During the study period, a total of 38 women were eligible for inclusion. There were 18 women who underwent mini-TLH + MC and were considered as the study group, while 20 women treated with mini-TLH were considered as the control group. The mean follow-up period of patients was around 29 (25–38) months. Demographic data were similar in both groups (Table 1).

The mean operation time was slightly longer in the mini-TLH + MC group than in the mini-TLH group, but the difference was not statistically significant. The mean blood loss was approximately 150 cc in the mini-TLH + MC group and 120 cc in the mini-TLH group. There was no statistically significant difference in terms of blood loss during operations in both groups. Vaginal cuff closure via the transvaginal route was performed with a significantly shorter operation time (7.6 ± 2.5 min; p = 0.002) than with the laparoscopic cuff closure (15.6± 7.1 min).

Apical vaginal support difference was improved by 2.2 cm (p = 0.027) at one year after mini-TLH + MC. The mean difference in C point was 1.03 cm in the mini-TLH group at one year after surgery, but this difference was not significant. Apical support changes at two years after operation were more prominent in the mini-TLH group (0.5 ± 0.6 cm) than in the mini-TLH + MC group (1.9 ± 1.2 cm, p = 0.03). The patients' verbal satisfaction rates postoperatively were similar between the mini-TLH and mini-TLH + MC groups and did not change over time in both groups (Table 2). The TVL values obtained immediately preoperatively and at one and two years postoperatively were 10.1 ± 3.7 cm, 9.8 ± 2.9 cm, and 9.4 ± 3.1 cm in the mini-TLH + MC group, respectively. The differences between the preoperative and postoperative TVL were not statistically significant. These results were also comparable with those in the mini-TLH group (Table 2).

The postoperative complications in the mini-TLH + MC group were febrile morbidity caused by cuff cellulites in one patient and cuff hematoma in another patient. One patient presented with bladder injury during operation, which required laparoscopic suturing. There was one case of febrile morbidity in the mini-TLH group. The complication rates were similar in both groups.

## 4. Discussion

To the best of our knowledge, this is the first attempt to address early results of the preventive potential of MC as an apical support procedure in addition to mini-TLH. In this study, the differences and probable advantages of performing MC in addition to mini-TLH were expressed in patients who did not have prolapse symptoms. MC was also executed in accordance with the patients' preference.

The mean difference between the pre- and postoperative measurements of the C point in the mini-TLH + MC group was very small and could be ignored after two years postoperatively. Conversely, in the mini-TLH group, the difference between the pre- and postoperative levels reached almost 50% of the preoperative level. Because this vaginal descent may increase further over time, we plan to continue following up these patients and evaluating the long-term results of this study. There was no significant difference in terms of TVL in the both groups with postoperative satisfaction.

Hysterectomy is one of the most common gynecological procedures for the treatment of benign uterine diseases worldwide [3, 13]. Performing hysterectomy by a laparoscopic approach has several benefits over the traditional abdominal technique [14], and thus, this method has been increasingly adopted over the years [3, 15]. Mini-laparoscopy is defined as surgery with instruments that are ≤ 3 mm in diameter, with the only possible exception of using 5-mm-diameter optics at the umbilicus. With the goal of reducing the overall scar burden and tissue trauma, increasing accuracy and precision, and possibly decreasing somatic pain, mini-laparoscopy has been used to perform procedures of ever-increasing complexity, encompassing an ever-growing range of surgical specialties [16, 17].

Vaginal apical suspension procedures include the uterosacral vaginal vault suspension, sacrospinous ligament fixation, iliococcygeus fascia suspension, and MC or Mayo culdoplasty [18]. MC is a technique performed via the vagina at the time of hysterectomy for prevention of apical prolapse or after hysterectomy to treat it. Laparoscopic MC was described by Ricci et al. via laparoscopic-assisted vaginal hysterectomy [19]. Although commonly performed, data describing the outcomes of MC as a preventive measure after mini-TLH are limited [6] or absent. There are limited studies evaluating the effects of MC on TVL and sexual function in the literature [20, 21]. A recent study reported that the MC shortens TVL postoperatively [21]. However, their patients were all postmenopausal, unlike ours. This result may be explained by mainly the urogenital atrophy usually present in the postmenopausal stage. All studies, including ours, revealed that the satisfaction rate postoperatively was not influenced by the vaginal length.

Risk factors for apical prolapse include vaginal deliveries, obesity, and previous hysterectomy. However, genetic predisposition leading to reduced connective tissue and muscle strength may also play an important role in the development of this condition [22]. The failure of MC could be seen more often in the patients who have undergone vaginal hysterectomy with a history of vaginal delivery of macrosomic infants [23]. Despite there being expectations of reduced apical prolapse, this is controversial in TLH because of the lack of data in the literature to prove it. Because the colpotomy incision is made above the sacrouterine and cardinal ligament complex, this expectation is logical. Nevertheless, it has yet to be clarified whether this expectation is true. Therefore, the effect of TLH on the prevalence of vaginal cuff prolapse postoperatively will continue to be a topic of ongoing debate.

A recent study retrospectively evaluated MC culdoplasty and laparoscopic uterosacral plication performed alongside hysterectomies in order to prevent PHVP. They were found to be comparable in terms of the complications encountered. Laparoscopic uterosacral plication has a statistically significant shorter hospital admission, but MC has proven to be superior to laparoscopic uterosacral plication in terms of patients presenting with subsequent pelvic organ prolapse [24]. Another study reported that laparoscopic uterosacral ligament colpopexy appears to achieve a good anatomical outcome and is safer and more reliable than the transvaginal approach [25].

Closure of the vaginal cuff can be performed either vaginally or abdominally. Laparoscopic vaginal cuff closure is associated with some difficulties in terms of technical surgical aspects. For instance, this procedure requires agility with laparoscopic suturing. In mini-TLH, it is difficult to take a strong “bite” with a 3-mm needle holder during laparoscopic cuff closure, thus resulting in increased operation time. In our study, laparoscopic cuff closure took approximately 15 minutes, whereas vaginal cuff closure took approximately 7 minutes. Consequently, we consider that the vaginal cuff closure by the transvaginal route in addition to mini-TLH is an easy and quick procedure. Furthermore, vaginal cuff closure by the transvaginal route allows the operator to perform the apical support procedure concomitantly.

Vaginal vault dehiscence after a hysterectomy is rare, but it is a possibly life threatening condition. Most cases occur after total hysterectomy. TLH may be associated with an increased risk of vaginal vault evisceration [26, 27]. Because laparoscopy is increasingly being used instead of abdominal hysterectomy, it is important to be aware of this complication. A recent study reported that transvaginal closure at the end of a TLH is associated with a significantly higher incidence of both vaginal dehiscence and any vaginal cuff complication [28]. In mini-TLH, laparoscopic cuff closure may cause vaginal cuff dehiscence because of the widespread use of energy, technical limitations, and difficulties in suturing. Therefore, it seems more logical to perform a cuff closure by a vaginal approach than by laparoscopic cuff closure.

The main limitations of our study may be the retrospective design and small sample size. In addition, patients were evaluated over a relatively short period of time for prolapse and were not evaluated with the FSFI questionnaire before surgery. However, our study does have several strengths, such as being the first to report on the outcomes of mini-TLH with or without the apical support procedure performed by a single senior operator. Furthermore, the POP-Q evaluation was performed by the same urogynecologist, and we included a homogenous group of patients with pelvic organ prolapse in this study.

In conclusion, prophylactic MC with a vaginal approach during mini-TLH appears to be a safe, simple, and efficient procedure to prevent apical prolapse without severe morbidity and extra burden for patients. Further prospective, randomized, controlled trials are warranted to better define the preventive potential of MC in addition to mini-TLH.

## Figures and Tables

**Table 1:** Demographic data of women undergoing mini-TLH with MC and only mini-TLH.

Parameters	Mini-TLH + MC	Mini-TLH	P value
	(n = 18)	(n = 20)	

Age (years)	45.3 ± 6.9	44.1 ± 5.8	0.48
Gravidity (median, min - max)	3 (0 - 3)	4 (0 - 4)	0.24
Parity (median, min - max)	2 (0 - 3)	3 (0 - 5)	0.35
BMI (kg/m^2^)	24.6 ± 2.5	25.1 ± 4.8	0.17
Mean follow-up period (months)	28.4 ± 4.2	30.1 ± 6.7	0.28

*Mini-TLH*: mini total laparoscopic hysterectomy, *MC*:McCall culdoplasty,* BMI*: Body mass index.

Values presented as mean ± SD, unless stated otherwise.

**Table 2:** Comparison of main outcome measures between groups.

Parameters	Mini-TLH + MC	Mini-TLH	P value
	(n = 18)	(n = 20)	

Operation duration (min)	86.2 ± 20.4	74.3 ± 15.2	0.24
Mean blood loss (cc)	150.4 ± 40.9	120.6 ± 50.4	0.36
Cuff closure time (min)	7.6 ± 2.5	15.6 ± 7.1	0.002∗
Complication rate (n, %)	1 (5.6%)	1 (5%)	0.78
Apical support differences after one year (cm)	2.2 ± 1.4	1.03 ± 0.7	0.027∗
Apical support differences after two years (cm)	1.9 ± 1.2	0.5 ± 0.6	0.03∗
Total vaginal length preoperatively (cm)	10.1 ± 3.7	11.2 ± 3.5	0.065
Total vaginal length after one year (cm)	9.8 ± 2.9	10.2 ± 2.8	0.68
Total vaginal length after two years (cm)	9.4 ± 3.1	9.6 ± 2.9	0.71
Satisfaction rate after one year (%)	88.8	85	0.76
Satisfaction rate after two years (%)	94.4	85	0.66

*Mini-TLH*: mini total laparoscopic hysterectomy, *MC*:McCall culdoplasty, ∗ *p ≤* 0.05.

Values presented as mean ± SD, unless stated otherwise.

## Data Availability

The data used to support the findings of this study are available from the corresponding author upon request.
